# Multi Reflection of Lamb Wave Emission in an Acoustic Waveguide Sensor

**DOI:** 10.3390/s130302777

**Published:** 2013-02-27

**Authors:** Martin Schmitt, Sergei Olfert, Jens Rautenberg, Gerhard Lindner, Bernd Henning, Leonhard Michael Reindl

**Affiliations:** 1 Institute of Sensor and Actuator Technology, Coburg University of Applied Sciences, Am Hofbrauhaus 1, 96450 Coburg, Germany; E-Mail: lindner@hs-coburg.de; 2 Measurement Engineering Group, Faculty of Electrical Engineering, Computer Science and Mathematics, University Paderborn, Warburger Str. 100, 33098 Paderborn, Germany; E-Mails: olfert@emt.upb.de (S.O.); rautenberg@emt.upb.de (J.R.); henning@emt.upb.de (B.H.); 3 Laboratory for Electrical Instrumentation, Department of Microsystems Engineering-IMTEK, University Freiburg, Georges-Kohler-Allee 106, 79110 Freiburg, Germany; E-Mail: reindl@imtek.uni-freiburg.de

**Keywords:** multi reflection, lamb wave, emission, acoustic waveguide sensor, surface acoustic waves, leaky lamb waves, schlieren imaging

## Abstract

Recently, an acoustic waveguide sensor based on multiple mode conversion of surface acoustic waves at the solid—liquid interfaces has been introduced for the concentration measurement of binary and ternary mixtures, liquid level sensing, investigation of spatial inhomogenities or bubble detection. In this contribution the sound wave propagation within this acoustic waveguide sensor is visualized by Schlieren imaging for continuous and burst operation the first time. In the acoustic waveguide the antisymmetrical zero order Lamb wave mode is excited by a single phase transducer of 1 MHz on thin glass plates of 1 mm thickness. By contact to the investigated liquid Lamb waves propagating on the first plate emit pressure waves into the adjacent liquid, which excites Lamb waves on the second plate, what again causes pressure waves traveling inside the liquid back to the first plate and so on. The Schlieren images prove this multi reflection within the acoustic waveguide, which confirms former considerations and calculations based on the receiver signal. With this knowledge the sensor concepts with the acoustic waveguide sensor can be interpreted in a better manner.

## 1. Introduction

Sensors and actuators based on surface acoustic waves at the solid-liquid interface are the focus of much current research and development because of their high sensitivity and the high possibilities of miniaturization [1–5]. Typically, surface acoustic wave sensors and actuators are fabricated on a piezoelectric substrate, e.g., quartz, lithium niobate (LiNbO_3_) or lithium tantalate (LiTaO_3_) by micro machining techniques. Contrary to this trend, surface acoustic wave sensors and actuators can be realized on non-piezoelectric substrate, e.g., glass, aluminum or steel, in macroscopic dimensions of several millimeters [4]. In particular existing technical structures such as bearings, tiles or glass plates can be provided with sensors by the utilization of surface acoustic waves [6–8].

Recently, a new acoustic waveguide sensor based on multiple mode conversion of surface acoustic waves at the solid–liquid interfaces has been introduced for the concentration measurement of binary and ternary mixtures, liquid level sensing, the investigation of spatial inhomogenities and bubble detection [9,10]. The sound waves within the waveguide structure, which consists of two parallel plates equipped with a transmitter and a receiver, respectively, is assumed to propagate along a zigzag pathway [9,10]. This assumption was corroborated by the shape of the receiver signals consisting of several well distinguished wave groups and a simplified model simulation [9–12].

In this contribution Schlieren images of the sound propagation within such a waveguide sensor are presented the first time. The transmitter is excited by a continuous as well as a five cycles sinusoidal burst signal and stroboscopic measurements of the density variations in the liquid are made with a laser-based Schlieren set-up at different points in time.

## 2. Acoustic Waveguide Sensor

**Figure 1.**
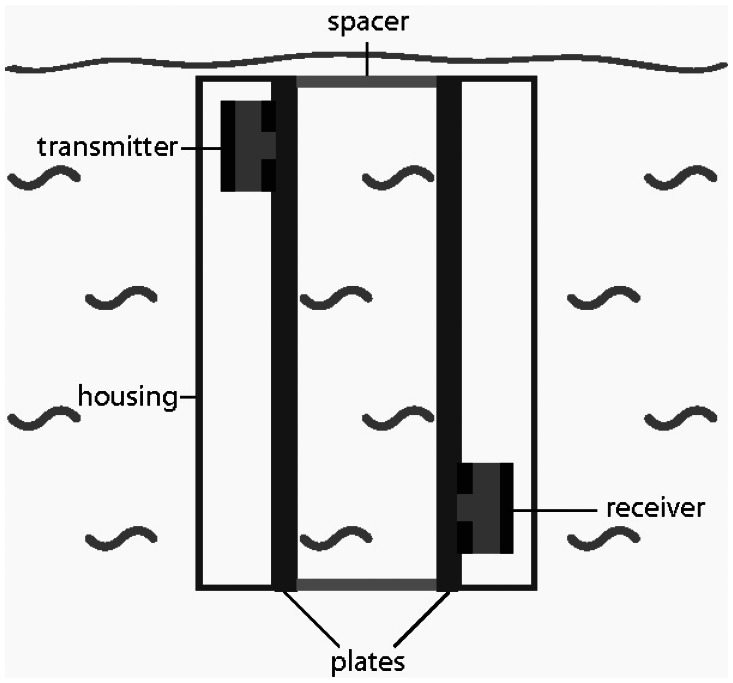
The acoustic wave guide sensor is dipped into the liquid in such a way that the investigated liquid is in the gap between the two plates.

**Figure 2.**
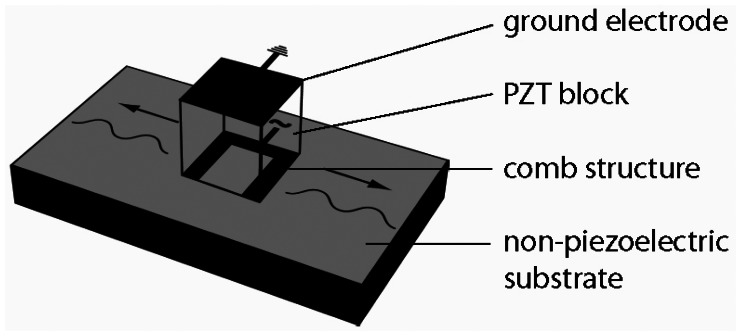
Single phase transducer for exciting surface acoustic waves on non-piezoelectric substrate.

Two parallel plates (100 mm × 40 mm × 30 mm) equipped with single phase transducers on the exterior side are used to build up the acoustic wave guide sensor (Figure 1). The single phase transducer consists of an electrically polarized polycrystalline lead zirconium titanate (PZT) block (6 mm × 4 mm × 1 mm), which is structured by screen printing with copper-nickel electrodes of 10 μm thickness (Figure 2). The upper side of the block is fully metalized, but on the lower side the coating forms a two finger comb structure. On the upper electrode the signal ground and on the lower electrode the high frequency signal is applied. One single phase transducer acts as transmitter the other as receiver in the acoustic waveguide configuration (Figure 1). Spacers are holding the plates in a constant distance of 12 mm. In order to protect the single phase transducers from liquid, that side of each plate is covered by a housing. The acoustic waveguide sensor is tipped into the liquid, whereas the gap between the two plates is brimmed by the liquid (Figure 1).

**Figure 3.**
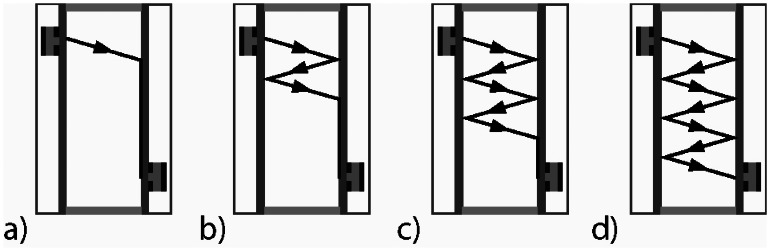
The propagation path of the emitted pressure wave into the liquid by Lamb wave excitation on the emitter plate forms a zigzag pathway (**a**–**d**), by a combination of a repeated pressure wave emission into the liquid from Lamb wave propagation on the plate and a repeated Lamb wave excitation on the other plate caused by the incidence of pressure waves from the liquid.

**Figure 4.**
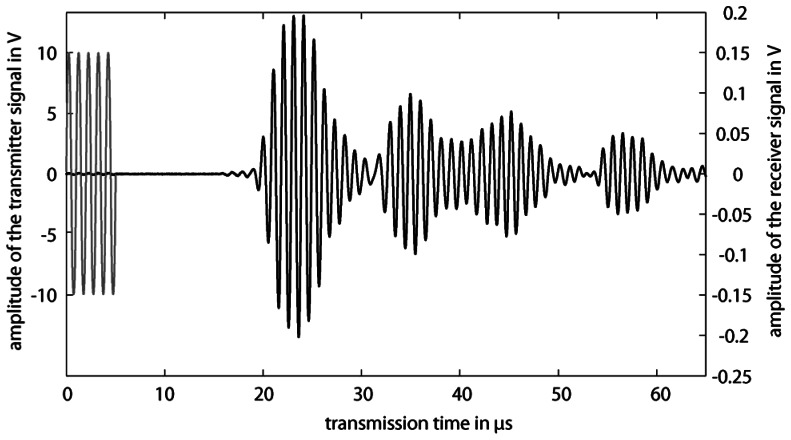
The excitation signal of the acoustic waveguide sensor is a sinusoidal burst with five cycles (**grey**). The receiver signal consists of several distinguished wave groups (**black**).

Lamb waves are excited and detected on the plates by single phase transducers, which radiate a pressure wave under a characteristic angle, called Lamb angle, into the liquid [10]. On the emitter plate a Lamb wave is excited by the single phase transducer. Due to mode conversion a pressure wave is emitted into the liquid. This pressure wave excites on the receiver plate a Lamb wave again, which is propagating on the plate to the receiver (Figure 3(a)). During the propagation on the plate, a pressure wave will be radiated into the liquid. On the emitter plate, this pressure wave will be converted into a Lamb wave and again radiated as another pressure wave back into the liquid. This pressure wave will be converted into a Lamb wave on the receiver plate, which will radiate a further pressure wave into the liquid again and the residual Lamb wave on the receiver plate will be detected by the receiver (Figure 3(b)). This phenomenon will be repeated a few times in such a way that the wave propagation will build up a zigzag pathway in the gap between the two plates (Figure 3(c,d)). Thus an acoustic waveguide with bulk waves in the liquid will be built up [13]. By burst operation of the emitter, the receiver signal consists of several distinguished wave groups, from which the transmission time of each wave group is evaluated by determination of a predefined zero crossing and the absolute amplitude of each wave group is measured peak to peak (Figure 4). Using the acoustic waveguide sensor concentration measurement of binary and ternary mixtures with integrated temperature compensation, liquid level sensing, investigation of spatial inhomogenities and bubble detection can be realized [6–9,14].

## 3. Measurement Setup

**Figure 5.**
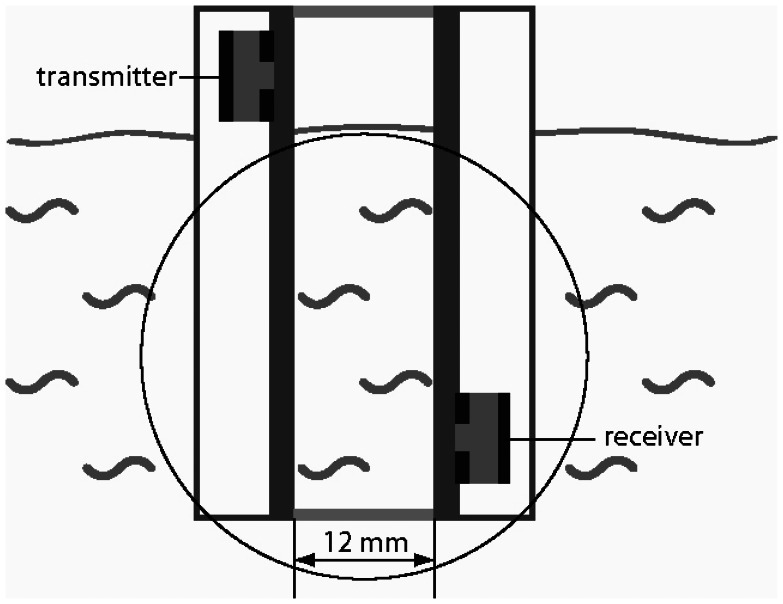
The measurement setup consists of two parallel glass plates at a distance of 12 mm. The acoustic wave guide is dipped into the water in such a way, that the emitter is above the liquid level. The circle marks the inspection glasses of the Schlieren set-up.

The acoustic waveguide sensor setup for the Schlieren measurements consists of two parallel glass slides with a thickness of 1 mm and a distance between the plates of 12 mm. The waveguide sensor is dipped into water in such a way, that the transmitter is above the liquid level (Figure 5). The transmitter and the receiver are single phase transducers with a center frequency of 1 MHz, which are designed to excite dominantly the antisymmetrical zero order Lamb wave mode on thin plates [15]. The transmitter is excited either by a continuous sinusoidal signal or a burst sinusoidal signal of 5 cycles and 32 Vpp amplitude. Schlieren images are taken after starting the excitation signal of the transmitter at different time steps.

**Figure 6.**
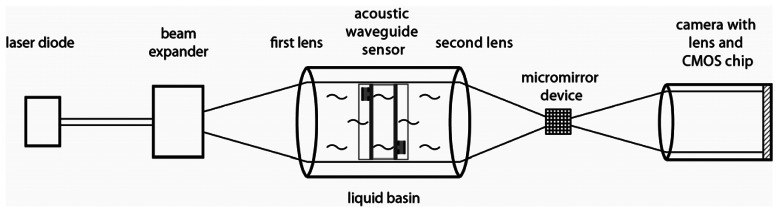
Measurement setup for the Schlieren system.

The Schlieren set-up consists of a pulsed diode laser, a water filled basin, a digital micromirror device, a CMOS chip and optical lenses (Figure 6). The diode laser acts as a point light source. The pulsed laser light is parallelized by the first lens, passes through the water basin and is refocused by the second lens. In the focus of the second lens, also called Fourier plane, the light is high-pass filtered by the digital micromirror device. Form the digital micromirror device the light is projected on the CMOS chip by the lens of the camera. For the measurements the acoustic waveguide sensor is positioned in the liquid basin in such a way, that the sound wave propagation within the waveguide sensor is normal with respect to the light propagation direction. Sound wave propagation within the liquid results in a change of local refraction index for the liquid and therewith the phase of the light will be changed. By high-pass filtering in the Fourier plane the change of the phase is transferred into a change in amplitude [16]. The depicted amplitude in the Schlieren images is connected with the square value of the sound wave amplitude to due high-pass filtering. By pulsed operation of the laser diode the sound wave propagation within the liquid is stroboscopic recorded. More detailed information about the Schlieren system can be found in reference [17].

## 4. Measurement Results

**Figure 7.**
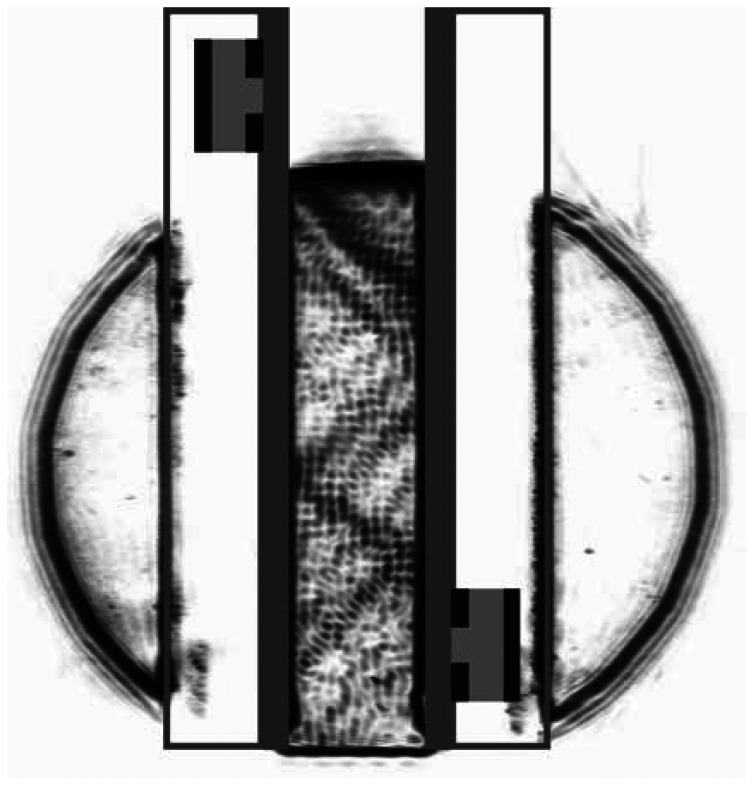
Schlieren image of sound propagation within the acoustic waveguide by continuous sinusoidal excitation of the transmitter. A zigzag propagation way can be observed. The location of the transducers and the plates are marked by a sketch.

At first the transmitter on the first glass slide is operated with a continuous sinusoidal excitation signal. Because the transmitter is above the liquid level, the excited Lamb wave enters into the liquid and emission occurs therein. This means that an inhomogeneous pressure wave is excited into the liquid at a characteristic angle, called Lamb angle. The pressure wave passes the liquid and is reflected back into the liquid again by the second glass slide (Figure 7). This process of passing and reflection occurs a few times within the liquid gap between the two plates. In the Schlieren image the sound propagation way through the liquid can be described by a zigzag pathway. In the first reflection on the right plate, a displacement of the outgoing reflected pressure wave can be observed. This displacement of the non-specular reflection corresponds to the Schoch phenomenon and is attributed to Lamb wave excitation and subsequent emission of pressure waves [18,19]. At the other places of reflection no such displacement can be seen. A possible explanation of this phenomenon can be provided by the assumption that besides the sound waves within the liquid Lamb waves are propagating on both plates and thus the plates may not be considered to be undisturbed except of the first reflection at the receiver plate.

**Figure 8.**
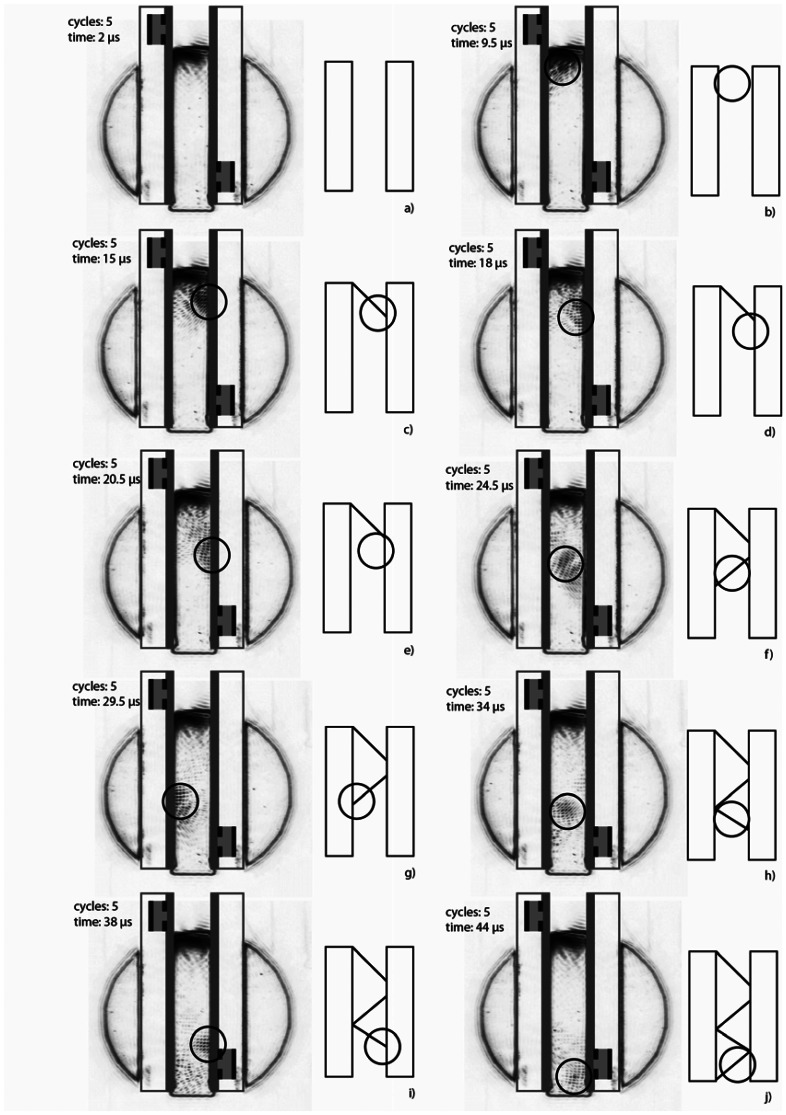
Schlieren images and sketches of sound propagation within the acoustic waveguide by 5 cycles burst excitation of the transmitter. The acoustic wave group is marked by a circle. The recording times are 2 μs (**a**), 9.5 μs (**b**), 15 μs (**c**), 18 μs (**d**), 20.5 μs (**e**), 24.5 μs (**f**), 29.5 μs (**g**), 34 μs (**h**), 38 μs (**i**) and 44 μs (**j**). The location of the transducers and the plates are marked by a sketch.

When the transmitter is operated with a sinusoidal burst signal of five cycles, a wave group following a zigzag pathway through the waveguide configuration can be observed in the Schlieren images at different points in time (Figure 8). Each reflection at the receiver plate produces a Lamb wave group on the plate, which radiates into the liquid again due to the emission process. Since the vertical distance between the transmitter and the receiver is only a few millimeters and the mode emission coefficient at the frequency thickness product of 1 MHz × 1 mm is about 90 Neper/m [20] a residual part of the excited Lamb wave group can be detected by the receiver. Because of the distance of the two plates (12 mm), the slow sound velocity of water (1480 m/s) and the fast Lamb wave velocity (2,210 m/s for 1 MHz × 1 mm) the wave groups of the receiver signal are separated in the time domain [10–12,15].

## 5. Discussion and Conclusion

With the application of Schlieren images the assumed zigzag sound propagation pathway through the liquid gap between the two plates of the acoustic waveguide has been proven. Moreover, the propagation of an acoustic wave group in a zigzag pathway through the waveguide structure by burst excitation confirms former explanations of the formation of the receiver signal wave groups by multi reflection. In the Schlieren figures a non-specular reflection of the first, undisturbed reflection location has been observed. The interpretation of additional interference pattern in the Schlieren images of the waveguide structure needs further investigations. The Schlieren images confirm the interpretation of measurement data and calculation results that the longer propagation way through the liquid by the zigzag pathway leads to a higher sensitivity with respect to changes in the liquid mixture [9]. Moreover, the spreading of one wave group to increasing numbers of wave groups with increasing immersion depth for liquid level sensing [9], the change of the measured transmission time of one wave group by immersion the acoustic waveguide sensor in stratified oil layer on water [9] or the influence of gas bubbles within the acoustic waveguide sensor [10] can be interpreted in a better manner.
